# An ICP-MS-Based Analytical Strategy for Assessing Compliance with the Ban of E 171 as a Food Additive on the EU Market

**DOI:** 10.3390/nano13222957

**Published:** 2023-11-15

**Authors:** Francesca Ferraris, Carlos Adelantado, Andrea Raggi, Sara Savini, Mohammed Zougagh, Ángel Ríos, Francesco Cubadda

**Affiliations:** 1 National Reference Laboratory for Nanomaterials in Food, Department of Food Safety, Nutrition and Veterinary Public Health, Istituto Superiore di Sanità—National Institute of Health, 00161 Rome, Italy; francesca.ferraris@iss.it (F.F.); andrea.raggi@iss.it (A.R.); sara.savini7@gmail.com (S.S.); 2 Flemish Institute for Technological Research (VITO), 2400 Mol, Belgium; carlos.adelantadosanchez@vito.be; 3 Department of Analytical Chemistry and Food Technology, University of Castilla-La Mancha, 13071 Ciudad Real, Spain; mohammed.zougagh@uclm.es (M.Z.); angel.rios@uclm.es (Á.R.); 4 Regional Institute for Applied Scientific Research, IRICA, 13005 Ciudad Real, Spain

**Keywords:** titanium dioxide, E 171, ICP-MS, single-particle ICP-MS, spectral interferences

## Abstract

A method was developed for the determination of total titanium in food and food supplements by inductively coupled plasma mass spectrometry (ICP-MS) after microwave-assisted acid digestion of samples. Five food supplements, including one certified reference material, and 15 food products were used for method development. Key factors affecting the analytical results, such as the composition of the acid mixture for sample digestion and the bias from spectral interferences on the different titanium isotopes, were investigated. Resolution of interferences was achieved by ICP-MS/MS with ammonia adduct formation and viable conditions for control laboratories equipped with standard quadrupole instruments were identified. The method was successfully validated and enables rapid screening of samples subject to confirmatory analysis for the presence of TiO_2_ particles. For the latter, single-particle ICP-MS (spICP-MS) analysis after chemical extraction of the particles was used. The two methods establish a viable analytical strategy for assessing the absence of titania particles in food products on the EU market following the E 171 ban as a food additive.

## 1. Introduction

Food-grade titanium dioxide, known as E 171 in the EU, is a widely used food additive which, owing to the light-scattering effect of TiO_2_ particles occurring in the particle size range of 200–300 nm, is used as a whitener [1]. Several studies have characterized the physicochemical properties of E 171 either as a pristine material [2,3,4,5,6,7,8,9,10] or as found in food samples [7,9,11,12,13,14,15,16], using transmission or scanning electron microscopy (TEM, SEM), coupled with energy dispersive X-ray spectroscopy (EDX) when food matrices are analyzed, for assessing the constituent particle size, and X-ray Diffraction (XRD), for assessing the crystalline phase. These investigations showed that E 171 appears as a polydisperse material composed by anatase (rarely rutile) particles, with a constituent size typically ranging 30–350 nm and smaller or larger particles being sparingly present. Recent TEM-based investigations documented that the fraction of constituent particles with a minimum external dimension < 100 nm lies in the range 18–82% by number and 2–41% by mass, with ca. half of the particles being below this threshold on average [10,15,17].

In 2021, the European Food Safety Authority (EFSA) completed a re-assessment of E 171 safety as a food additive based on the available body of evidence concerning TiO_2_ nanomaterials with constituent particles >30 nm and food-grade titanium dioxide [18]. Along with a potential for accumulation, immunotoxicity, inflammation, induction of aberrant crypt foci (by E 171) and neurotoxicity (by nanoparticulate forms), possible induction of DNA strand breaks and chromosomal damage by TiO_2_ particles was identified, with no certainty about a threshold mode of action. The concern for genotoxicity and the many uncertainties led to the conclusion that E 171 could no longer be considered safe, which in 2022 led to regulatory actions resulting in the ban of E 171 as a food additive in the EU [19]. The ban concerns E 171 in food and food supplements, whereas its use in toothpaste and oral medicines is, for the time being, not affected.

Testing compliance of food products on the EU market with the E 171 ban poses significant challenges to official control laboratories. Whereas TEM or SEM analysis coupled with elemental analysis (by, e.g., EDX) represents the confirmatory approach providing certainty of E 171 presence along with the capability to characterize the size of the constituent particles and their number-based size distribution (PSD), single-particle inductively coupled plasma mass spectrometry (spICP-MS) has emerged as an ideal technique for E 171 analysis with widespread availability in testing laboratories, higher sample throughput, and lower analytical costs [4,9,10,15,20,21,22,23,24,25,26]. In spICP-MS, suspended individual constituent particles and agglomerates (i.e., ‘secondary particles’ formed by two or more constituent particles) are concurrently sized. Several studies demonstrated that, when dispersion protocols minimizing particle agglomeration are used, the E 171 number-based PSDs from spICP-MS are similar to the constituent PSDs obtained by TEM or SEM [10,14]. Moreover, when E 171 particles are extracted by food matrices, they have been generally shown to be fairly de-agglomerated, e.g., owing to steric stabilization by adsorbed proteins and other food components, and also in this case the number-based PSDs obtained by spICP-MS and EM tend to overlap [9,15,24]. Owing to these desirable features, particle size analysis of E 171 by spICP-MS has been interlaboratory tested among seven experienced European laboratories with a successful outcome [9]. Only in complex matrices, e.g., dairy products rich in calcium (which causes spectral interferences affecting several Ti isotopes, see Table 1), the use of more complex ‘triple-quadrupole’ or high-resolution spICP-MS may be necessary [14].

Although spICP-MS is a viable means to assess the presence of E 171 in food products, efficient screening methods are needed to identify samples to be tested by this particle- and elemental-specific method. Determination of the total titanium concentration by ICP-MS appears as a practicable option and is investigated in the present study. The foundation of such an approach is the relatively low and uniform Ti concentrations in food, which in the first place depend on the low mobility and availability of the element in agricultural systems. Titanium is not an essential element for plants [27] or animals [28]. Titanium minerals are very resistant to weathering in soils and the titanium released precipitates as anatase; only upon dissolution, the soluble inorganic or organometallic species are sparingly taken up by edible plants [29]. Like most transition elements, root-absorbed Ti is largely accumulated in the roots with a small amount transported to shoots through the xylem stream [30]. Ti-containing commercial fertilizers used as biostimulants for improving crop production are available [31], but they are unlikely to result in substantially increased Ti concentrations in edible parts of the plant since, even for foliar-applied Ti, unidirectional translocation from shoots into roots has been observed [30]. In recent years, a few studies have claimed beneficial effects of agricultural applications of TiO_2_ particles [32,33], equaled by studies reporting detrimental effects [34,35]; however, the use of TiO_2_ is not established and, in addition, available evidence shows limited plant uptake [36].

Other than use of TiO_2_ food additives, food processing is also not expected to markedly contribute to total Ti levels in products. Titanium is used in certain so-called “stabilized” forms of stainless steels, which in general contain less than 1% Ti, and in some alloys, but negligible migration occurs owing to surface passivation [37]. Titanium dioxide is used in other food contact materials (e.g., paints, lacquers, enamels, paper-coatings and plastics), whereas titanium compounds are used as catalysts in the manufacture of plastics; associated migration is expected to be generally minimal.

Reliable analytical data on total Ti in food are limited. The element is rarely investigated and most of the historical data have limitations. Titanium is the ninth most abundant element in the earth’s crust and the second most abundant transition metal after iron; as a consequence, one general issue when dealing with background concentrations is positive contamination during sample preparation [38]. In recent years, ICP-MS has become the most widespread technique for elemental analysis, but all Ti isotopes are affected by severe spectral interferences arising from isobars and polyatomic ions (Table 1).

This means that, at background concentrations in food items, only data obtained by quadrupole mass spectrometers with reaction cells, ‘triple-quadrupole’ and high-resolution instruments can be considered generally reliable. Such data suggest that in most food, background Ti concentrations are generally <0.5 µg g^−1^ and can be as small as <0.05 µg g^−1^ [39].

Table 2 summarizes analytical Ti data from surveys on E 171 in food from the EU market before the ban.

Typical data are in the range of hundreds or thousands of µg g^−1^, when expressed as total Ti; however, concentrations in the range of few µg g^−1^ or lower were also found. This is in agreement with use levels reported by food business operators, ranging 0.08–5480 µg g^−1^ [41] and 1.2–11,987 µg g^−1^ [42]. It is worth noting that, in addition to E 171, titania particles have been detected in food as a consequence of the use of pearlescent pigments [10,15,24,43]. These consist of mica (potassium aluminum silicate) platelets coated with titanium dioxide nanoparticles. However, such pearlescent pigments have not been subject to regulatory risk assessment and as such are not authorized [44]. Until this possibly happen, their use has to be considered illegal, which translates in the non-compliance of whatever manmade TiO_2_ particle found in food or food supplements on the EU market. From the analytical point of view, total Ti concentrations associated to titanium dioxide in pearlescent pigments may be relatively low (especially when mixed forms with iron oxides are used). Also, confirmation via spICP-MS can be challenging as individual particles detached from the mica layer can be as small as 15–20 nm (i.e., below the spICP-MS size LoD); however, small, detached aggregates are detectable and also very small mica fragments bearing the coating of aggregated TiO_2_ particles can (in principle) be aspirated into the ICP-MS.

The present study aims at developing an analytical strategy for assessing the absence of titania particles in food products on the EU market founded on total Ti determination via ICP-MS as screening method, followed by spICP-MS analysis as confirmatory method. A tailored sample preparation, i.e., microwave digestion resulting in solubilization of TiO_2_ particles for total Ti, and chemical extraction of TiO_2_ particles for spICP-MS analysis, was developed. 

## 2. Materials and Methods

### 2.1. Instrumentation

A Nexion 350D ICP-MS system (Perkin Elmer, Shelton, CT, USA) equipped with a Meinhard concentric nebulizer, a glass cyclonic spray chamber and a standard quartz torch (2.5 mm i.d) was used for E 171 characterization and quantification with the Syngistix™ Nano Application software v. 2.5 (Perkin Elmer, Shelton, CT, USA). Total titanium analyses were performed by means of an 8800 ‘Triple quad’ ICP mass spectrometer (Agilent Technologies Inc., Tokyo, Japan).

For the determination of total Ti in food and food supplements, samples were digested in an Ultrawave Single Reaction Chamber Microwave Digestion System (Milestone, Bergamo, Italy). For the determination of total Ti in food supplements samples, when HCl was added to the mineralization mixture, an ETHOS E (Milestone, Bergamo, Italy) was used instead. A Vial Tweeter ultrasonic device UP200St (Hielscher, Teltow, Germany) was used for particle dispersion via indirect sonication. Other pieces of equipment used were a Bandelin Sonorex RK 510 H ultrasonic water-bath, a Bandelin SONOPULS HD 3200 probe ultrasonic homogenizer (Bandelin, Berlin, Germany) for direct sonication, and a Stuart™ Flask Shaker SF1. An automatic RM100 agate mortar grinder (Retsch GmbH, Haan, Germany) was used to pulverize the food supplements pills.

### 2.2. Reagents and Materials

Ultrapure water was obtained from a Milli-Q Element purification system with 0.22 µm filters (Millipore, Molsheim, France). HNO_3_ 67–69% *v/v* (ultrapure grade, Carlo Erba, Rodano, Italy), HF 47–51% *v/v* (ultrapure grade, Carlo Erba, Rodano, Italy), HCl (ultrapure grade, Romil, Waterbeach, Cambridge, UK), and H_2_O_2_ 30% *v/v* (ultrapure grade, Sigma-Aldrich, Darmstadt, Germany) were used for microwave digestions. Analytical-grade tetramethylammonium hydroxide (TMAH) from Merck was used for alkaline extraction. For analytical quality control, NIST 3280 Multivitamin/Multielement tablets containing titanium dioxide, with a reference mass fraction of 5400 ± 300 µg g^−1^, was used as certified reference material and included in each analytical batch (Gaithersburg, MD, USA).

For spICP-MS measurements, the dissolved ionic gold standard (1 g L^−1^ in 5% HCl) and gold nanoparticles with a nominal diameter of 60 nm (43.45 μg mL^−1^ in aqueous 2 mM sodium citrate) were purchased from High-Purity Standards (Charleston, SC, USA) and NanoComposix (San Diego, CA, USA), respectively. The dissolved ionic titanium standard for ICP-MS (1 g L^−1^ in 2% HNO_3_) was purchased from High-Purity Standards (Charleston, SC, USA).

### 2.3. E 171-Containing Samples

The E 171-containing food and food supplements used in the present study, purchased from local markets, are described in Table S1.

### 2.4. Microwave-Assisted Acid Digestion of Food and Food Supplements

Sample treatment and analyses were performed in a clean-room facility. All food samples that required homogenization, were manually cut with a ceramic-blade knife into smaller pieces until obtaining a homogeneous sample. Subsamples (300 mg) were weighed and placed in high-pressure Teflon containers with 3 mL of HNO_3_ 67–69% *v*/*v*, 0.5 mL of H_2_O_2_ 30% *v*/*v*, and 0.2 mL of HF 47–51% *v*/*v* (all ultrapure grade). Samples were digested in the Ultrawave microwave system with the following program: (i) ramp from room temperature to 220 °C at 150 bar (23 min), (ii) constant temperature and pressure (10 min), and (iii) cooling down to room temperature and atmospheric pressure. Then, the samples were diluted to a final weight of 10 g with Milli-Q water and stored in fridge until total titanium analysis. A selection of food samples, listed in Table S2, were also digested in the same conditions but without HF.

Food supplements in tablet form were ground in order to obtain a homogeneous powder. Subsamples (300 mg) were weighed and placed in high-pressure Teflon containers with 1.5 mL of Milli-Q water, 5 mL of HNO_3_ 67–69% *v*/*v*, 0.75 mL of HF (all ultrapure grade). Samples were digested as described above. Then, the samples were diluted up to 30 g with Milli-Q water and stored in fridge until total titanium analysis. This procedure was applied also to SRM NIST 3280, after a batch of 15 tablets was ground for 10 min according to the supplier’s instructions. A selection of food supplement samples, listed in Table S2, were also digested in the same conditions but without HF.

Food supplements showing a visible residue after digestion were also submitted to the same procedure as above but with the addition of 1.5 mL of ultrapure HCl. In this case, the Microwave Digestion Labstation Ethos E was used with the following program: (i) ramp from room temperature to 120 °C (10 min), (ii) constant temperature (8 min), (iii) ramp from 120 °C to 190 °C (10 min), (iv) constant temperature (15 min) and then (v) cooling down to room temperature.

### 2.5. Alkaline Extraction of Particles from Food and Food Supplements

Subsamples (500 mg) of selected foods (*n* = 3) and food supplements (*n* = 4) were accurately weighed and 20 mL of 20% TMAH were added. The mixture was probe-sonicated for 5 min (ice bath; 50 W, pulse 6 + 2, MS72 probe). Afterwards, the dispersions along with two procedural blanks were placed into a mechanical agitator and stirred overnight. All samples were then diluted as needed for spICP-MS determinations.

### 2.6. Recovery Studies

A typical, well characterized food-grade anatase sample (referred to as ‘E 171-a’ in [45]) was dispersed (0.1 mg/mL) in water by bath sonication for 5 min, followed by indirect sonication by means of a Vial tweeter (amplitude 75%, cycle 0.5, time 25 min). Then, 1 mL of dispersion was added to a pre-tested E 171-free food and food supplement (4 aliquots each). The two TiO_2_ spiked samples were used, along with the certified reference material, to assess the recovery of total Ti after digestion (as described in 2.4) and ICP-MS determination. The food sample was submitted to digestion both with and without HF.

### 2.7. Total Titanium Analysis

For total titanium analyses, the Agilent 8800, which is equipped with an octopole-based collision/reaction (ORS3) cell in between the two-quadrupole analyzers, was operated in (i) the single quadrupole (SQ) mode and in the MS/MS mode either (ii) on-mass or (iii) in mass-shift mode with ammonia adduct formation. For the latter, a NH_3_/He mixture (1:9) was used as reaction gas to ensure interference-free Ti detection. The sample introduction system of the ICP mass spectrometer consisted in inert (non-quartz) components, i.e., a 2.5 mm ID sapphire injector, a PFA concentric nebulizer, and a double-pass PFA spray chamber cooled down to 2 °C. Instrument operating conditions are given in Table 3. 

The system was tuned daily for maximum Ti sensitivity and quantitative determinations were carried out by external calibration. After a preliminary study, two calibration curves were used, i.e., 0.25–0.5–1–5–10 µg L^−1^ for method blanks and low concentration samples, and 1–5–10–25–50 µg L^−1^ for samples with high titanium concentrations. All standards presented the same concentration of HNO_3_ (2%) (selected to mimic the acidic matrix of diluted samples) and internal standard, i.e., Rh (0.5 µg L^−1^).

### 2.8. Single-Particle ICP-MS

The operating conditions were optimized daily to achieve maximum sensitivity for titanium. Parameter setting and data acquisition were performed with the Nano Application Module of the Syngistix™ (2.5) software. The transport efficiency (TE) was determined daily following the ‘particle size approach’ [46] (the average value is shown in Table 4 along with the other instrumental parameters). For TE determination, the exact flow rate of the peristaltic pump was measured daily and was approximately 0.3 mL min^−1^ on average.

For the determination of the equivalent spherical diameter (ESD) of TiO_2_ particles, obtained from the particle mass assuming a spherical geometry, the Ti isotope at *m*/*z* 48 was monitored. A 4-point calibration curve ranging from 0 to 30 μg L^−1^ dissolved titanium was used. Immediately before analysis, samples were diluted with ultrapure water to achieve a particle concentration of 1000–2000 particles per 60 s scan time. The performance characteristics of the method are summarized in the SM. In spICP-MS measurements, the smallest pulse height that can be distinguished from the background determines the smallest detectable particle mass that can be related to the smallest detectable particle size. The instrumental size limit of detection (LoD) was calculated on a daily basis as the smallest particle distinguishable from the background and ranged from 27 to 39 nm.

## 3. Results

### 3.1. Total Titanium Analysis

Total titanium was determined using an ICP-tandem mass spectrometer that can be operated in either the SQ or MS/MS mode. The isotopes monitored on Q1 were ^46^Ti, ^47^Ti, ^48^Ti, ^49^Ti, ^50^Ti. However, only ^49^Ti and ^50^Ti were selected for method development in the SQ mode since, as shown in Table 1, the concentration values obtained were in most cases in good agreement with the ones obtained in the MS/MS mode, which provides the most effective solution to overcome spectral interferences. For instance, the most abundant ^48^Ti isotope, which is particularly sensitive to isobaric calcium interferes, delivered severely biased values with Ca-rich samples (e.g., the cappuccino powder, ice cream-based dessert and the white chocolate-containing food, see Table S3), highlighting its unsuitability as analytical mass.

With double mass selection in the MS/MS mode, in the first place the five Ti isotopes (*m*/*z* 46–50) were monitored in Q2 (the on-mass mode) and again ^49→49^Ti and ^50→50^Ti were selected for method development. Then, the mass-shift mode with the use of NH_3_/He as reaction gas was explored as more versatility in avoiding spectral overlaps is expected with this strategy, providing greater effectiveness in achieving interference-free conditions. The ammonium cluster product ions at *m*/*z* 149 and 150, i.e., Ti(NH_3_)_6_^+^ (47 → 149, 48 → 150), were selected for method development.

The method was validated over a period of several days. The results that follow refer to the procedure entailing digestion in presence of HF (see Section 3.1.1 and Section 3.1.2). Repeated measurements of SRM 3280 were used to establish precision, expressed as repeatability and reproducibility, which result to be <1.2% and <3.0%, respectively (Table S4). As an example, the values for the 47 → 149 mass shift are 1.1% and 2.9%, respectively. Recovery, determined by spiking samples of one food and one food supplement, is in the range 106–110% and 105–117%, respectively (see Section 3.1.2). The LoD (and in brackets, limit of quantification, LoQ) in the food matrix, based on the 3σ (6σ) criterion—where σ is the standard deviation of the measurement of 20 method blanks—were in the order of 0.04 µg g^−1^ (0.08 µg g^−1^) in mass shift and 0.07 µg g^−1^ (0.14 µg g^−1^) in SQ (^50^Ti) (Table S5). Method blanks (i.e., digestion reagents) were submitted to the complete analytical procedure and their Ti concentrations were always below the LoD in matrix.

Total titanium concentration values determined in the food supplements and food samples covered in the present study are reported in Table 5 and Table 6, respectively. In general, the six selected combinations of isotopes and detection modes provided almost identical results. For a few samples, occasional fluctuations were observed especially with ^50^Ti (SQ and on-mass MS/MS), but overall data remain fairly comparable. However, when low Ti levels have to be detected (i.e., few µg Ti g^−1^ or lower), the use of ammonia adducts at *m*/*z* 149 and 150 provides more accurate results because they enable better resolution of spectral interferences.

#### 3.1.1. Effect of Different Acid Mixtures for the Digestion of Food and Food Supplements

Since hydrofluoric acid is a hazardous reagent, oxidative digestion without HF was tested in order to assess to what extent its removal from the mixture of mineralization reagents affects the analytical results. As shown in Table 5 and Table 6, in samples containing E 171 at levels of hundreds or thousands of µg g^−1^, the detected titanium concentrations decrease dramatically (i.e., of an order of magnitude) in absence of HF.

As to HCl, for food supplements its addition may improve solubilization of visible residues when these are present, but in absence of HF no significant beneficial effect in solubilizing titanium and improving its detection is observed.

#### 3.1.2. Recovery Studies

The results of the recovery studies with E 171-spiked samples are shown in Table 7. In presence of HF, satisfactory recoveries are obtained for both the food and the food supplement samples tested. For the food sample, Ti recovery in the absence of HF in the mineralization mixture was also tested and turned out to be markedly reduced, confirming the results presented in Section 3.1.1.

The recoveries observed with respect to the certified Ti value of SRM 3280 are ca. 80% (Table 5). Also in this case, when HF is not used, recoveries drop of an order to magnitude (to ca. 5%). On the other hand, if the HF concentration in the digestion mixture is increased, the recovery improves up to 90% when 1.25 mL are added (Table S6); in this condition, the found value is not statistically distinguishable from the reference mass fraction when the respective uncertainties are taken into account. Interestingly, a higher HF concentration does not provide any improvement in the total Ti recovery.

### 3.2. Single-Particle ICP-MS

Results of spICP-MS analysis of selected food supplements (*n* = 4) and food samples (*n* = 3) are summarized in Table 8. Data indicate the presence of TiO_2_ particles in all the samples and the resulting Ti mass concentrations are in broad agreement with the independently measured total Ti concentrations by ICP-MS/MS, with TiO_2_ mass recoveries in the range 64–127%. In the four food supplements, E 171 was declared in the label and total Ti analysis confirmed this, with substantial amounts measured (total Ti levels > 1 mg g^−1^). In the food samples, total Ti levels were markedly lower. In the sample having E 171 declared on the label, the total Ti concentration was ca. 0.9 mg g^−1^. The other two samples, where E 171 was not mentioned on the label, presented much lower total Ti concentrations (i.e., ca. 2.5–5.0 µg g^−1^), but still exceeding the expected background levels in food. The number-based PSDs of these two samples differ from the typical E 171 PSDs found in the other five samples. The mean particle diameter is smaller and the fraction of particles < 100 nm is 49–54%, which is markedly higher than the range (15–28%) observed in the other samples. These characteristics appear to be compatible with the presence of pearlescent pigments containing titania. This would mean that the detected particles are likely aggregates of very small TiO_2_ particles. 

## 4. Discussion

The present study demonstrates the feasibility of using total Ti determination via ICP-MS after MW-assisted solubilization of TiO_2_ particles as screening method for testing compliance of food products on the EU market with the E 171 ban. Alkaline extraction of TiO_2_ particles followed by spICP-MS analysis was shown to be a rapid and efficient confirmatory method, which enables to characterize the number-based PSDs.

When E 171 levels in a food product are in the order of magnitude of several tens of µg g^−1^ as total Ti, all Ti isotopes can be used to screen positive (i.e., E 171-containing) samples by ICP-MS. However, E 171, as well as the equally illegal TiO_2_-containing pearlescent pigments, may be present at lower levels in food products. At these levels, the signal of the three major Ti isotopes may be heavily biased by spectral interferences resulting in apparent concentrations that do not reflect actual Ti contents in the product. In these cases, the selection of the major Ti isotopes as analytical masses may provide inaccurate results which would lead to incorrect classification of samples (false positives). Collision cell approaches are not expected to be effective in overcoming these interferences.

In this study, we showed that use of the two minor Ti isotopes provides more accurate data and, in most cases, can provide a correct classification of positive samples even at levels of a few µg Ti g^−1^. Measurement of both isotopes is recommended. When deviations between the two are found or when low total Ti levels need to be quantified, resolution of interferences by means of quadrupole mass spectrometers with reaction cells, ‘triple-quadrupole’ or high-resolution instruments is mandatory. In these conditions, in addition to high accuracy, the detection power of ICP-MS and the LoDs achieved outperform any other readily available analytical technique. In particular, we showed that the use of NH_3_/He in a triple-quadrupole ICP-MS to detect the titanium/ammonia adducts in the mass-shift mode at *m/z* 149 and 150 provides accurate results and improves LoDs. Balcaen et al. have shown that, although higher signal intensities can be obtained using O_2_ as a reaction gas and determining Ti via the corresponding molecular ions, this approach is not capable of dealing with all possible interferences. In fact, not only Ti is converted into TiO^+^, but also many of the interfering ions undergo a similar reaction; the spectral interferences are thus not resolved, but just transferred to another mass region [47].

In the present work, we developed a digestion method in which minimal use is made of hydrofluoric acid to dissolve TiO_2_ particles, i.e., 200 µL in food samples and 750 µL in food supplements. This amount, although in large stoichiometric excess when compared to the titania contents of the samples we tested, might not be sufficient to entirely dissolve TiO_2_ particles if, e.g., other particles reacting with HF (such as silicates) are present. For instance, 80% recovery was observed in this study for total Ti in the food supplement certified reference material, which contained 5.4 mg Ti g^−1^, and the presence of a small sediment was noted after digestion. Nevertheless, mindful of the hazardous properties of HF, we tested to what extent its removal from the mixture of mineralization reagents affects the analytical results. The substantial decrease in Ti recoveries that was observed is most likely due to agglomeration of undissolved TiO_2_ particles and sedimentation in sample vials or in the sample introduction system of the mass spectrometer. This is supported by zeta potential curves for E 171, which show lower absolute values at very small pH, whereas alkaline conditions are best to obtain a stable dispersion of TiO_2_ particles [10]. If this shows that accurate quantitative results for total Ti can only be achieved with the use of HF, although in minimal amounts, it is nevertheless true that positive samples can be correctly screened even avoiding this acid in the digestion mixture. Levels of tens or hundreds of µg Ti g^−1^ (as detected after digestion without HF) unequivocally indicate that E 171 is present, whereas at lower concentrations total Ti measurements are not appreciably affected by HF use. At low concentrations, instead, other analytical considerations become crucial, namely the prevention of sample contamination. The collection of an extensive dataset of total Ti concentrations in method blanks allowed us to identify a substantial variability, exceeding that detected for most of the other trace elements. The absolute magnitude of the variability was mitigated in this study by the use of ultrapure reagents and clean room facilities. However, if such conditions are not met, it can be expected that a substantial worsening of the analytical LoDs (LoQs) occurs.

Compliance testing of food products on the EU market can be facilitated by prioritization strategies in the selection of samples. Consideration of the food categories in which E 171 was used before its ban is one criterion and this information is widely available (e.g., see Section 4.4.1 and appendix R in [18]). The analytical surveys on E 171 (see Introduction) are the other essential information sources. Among them, the recently published large-scale screening of E 171 on the French market over five years (2018 to 2022) is especially valuable [26]. This survey showed that particular attention should be addressed to food products imported on the EU market since they may contain food-grade TiO_2_ without necessarily mentioning it on the ingredient list. Indeed, 63% of the food products found to contain E 171 in 2022 were originating from non-EU countries [26].

Based on the present study, the major expected challenge in screening for the presence of manmade TiO_2_ particles in food is the detection of low but still suspicious Ti levels. Only four samples, among the several labelled as not containing E 171, were obviously negative in this study. At present, there is a lack of reliable data on background titanium concentrations in food. Titanium is a rarely investigated element and the analytical challenges highlighted in this study result in a limited number of surveys providing accurate values for naturally occurring Ti in food. When manmade TiO_2_ particles are present in food at relatively low concentrations (e.g., 2 µg g^−1^ or lower), which may happen because of their use in an ingredient (causing a dilution in the final product with respect to the much higher levels generally found), there are at present limitations in the use of total Ti to screen potentially positive samples to be submitted to confirmatory analysis by spICP-MS and/or EM with EDX detection. There is a need to improve the available dataset of naturally occurring titanium concentrations in food and document the existence of potential Ti-rich foods (if any) to reduce the uncertainty in such cases. Ideally, the dataset should also comprise, for each food, the characterization of the titanium speciation (soluble forms vs. particles of environmental origin).

Until such a dataset becomes available, in cases of uncertainty, a pragmatic approach that control laboratories can follow is to compare the total Ti content and the content and PSDs of TiO_2_ particles in the suspect sample with those of equivalent products on the market.

## 5. Conclusions

A method was developed and successfully validated for the determination of total Ti in food and food supplements by ICP-MS. It allows for rapid screening of samples subject to confirmatory analysis for the presence of TiO_2_ particles. Selected samples, either labelled as containing E 171 or not, were successfully submitted to particle-specific analysis by spICP-MS with µs-dwell times. The two approaches establish a viable analytical strategy for assessing the absence of titania particles in food products on the EU market following the E 171 ban as a food additive.

## Figures and Tables

**Table 1:** The isotopic abundances and interferences for each of the isotopes of Ti. The isotopic abundance for each of the isobaric interferences is shown in brackets.

Isotope	Abundance (%)	Isobaric Interferences	Polyatomic Interferences
^46^Ti	8.25	^46^Ca^+^ (0.004)	^32^S^14^N^+^, ^14^N^16^O_2_^+^, ^15^N_2_^16^O^+^
^47^Ti	7.44		^32^S^14^N^1^H^+^, ^30^Si^16^O^1^H^+^, ^32^S^15^N^+^, ^33^S^14^N^+^, ^15^N^16^O_2_^+^, ^14^N^16^O_2_^1^H^+^, ^12^C^35^Cl^+^, ^31^P^16^O^+^
^48^Ti	73.72	^48^Ca^+^ (0.187)	^32^S^16^O^+^, ^34^S^14^N^+^, ^33^S^15^N^+^, ^14^N^16^O^18^O^+^, ^14^N^17^N_2_^+^, ^12^C_4_^+^, ^36^Ar^12^C^+^
^49^Ti	5.41		^32^S^17^O^+^, ^32^S^16^O^1^H^+^, ^35^Cl^14^N^+^, ^34^S^15^N^+^, ^33^S^16^O^+^, ^14^N^17^O_2_^1^H^+^, ^14^N^35^Cl^+^, ^36^Ar^13^C^+^, ^36^Ar^12^C^1^H^+^, ^12^C^37^Cl^+^, ^31^P^18^O^+^
^50^Ti	5.18	^50^Cr^+^ (4.345), ^50^V^+^ (0.25)	^32^S^18^O^+^, ^32^S^17^O^1^H^+^, ^36^Ar^14^N^+^, ^35^Cl^15^N^+^, ^36^S^14^N^+^, ^33^S^17^O^+^, ^34^S^16^O^+^, ^1^H^14^N^35^Cl^+^, ^34^S^15^O^1^H^+^

**Table 2:** Analytical Ti data from surveys on E 171 in food from the EU market *^a^*.

Sample Type	*n ^b^*	Total Ti (mg g^−1^)	Ref.
Chewing gum	7	0.15–4.5	[7]
Softmints	1	0.66	[40]
Creamed horseradish	1	1.7	
Dressings	2	0.6–4.49	
Chewing gum	5	ca. 0.6–5.5	[4]
Toothpaste	3	ca. 2.8–4.9	
Mayonnaise	1	ca. 0.7	
Hard candy	2	ca. 0.4–2.5	
Chocolate	2	ca. 0.5–0.9	
Soft candy	3	ca. 0.15–0.5	
Pastry	3	ca. 0.1–0.4	
Button shaped candies	1	0.7	[15]
Chewing gum	2	1.51–1.6	
Cake decoration	4	0.64–0.89	
Marzipan and sugar paste	2	1.86–3.2	
Mints	2	1.16–3.73	
Confectionery	2	0.13–0.67	
Ice frosting	2	0.17–0.35	
Coated biscuit	1	0.31	
Instant powder orange drink	1	1.92	
Chocolate confectionery	2	0.87–1.36	
Croissant filling	1	4.04	
Toothpaste	1	0.57	
Acidic food matrix	86	<0.1–4.00	[26]
Food containing pearlescent colorant	13	<0.1–2.17	
Other food matrix	148	<0.1–11.46	
Sugar-coated candies	105	<0.1–4.90	

*^a^* For [26], the subcategories belonging to the sample descriptions above are as follows. *Acidic food matrix:* biscuit and pastry, dehydrated drink, drink, jelly and soft candies, lollipop and hard candy, pastry decoration, snack, spice; *Food containing pearlescent colorant:* alcoholic beverage, drink, pastry decoration, sauce, spice; *Other food matrix*: alcoholic beverage, biscuit and pastry, chewing gum, dehydrated drink, food supplement, intermediate ingredient, jelly and soft candies, lollipop and hard candy, pastry decoration, sauce, snack, spice, syrup; *Sugar-coated candies*: chewing gum, jelly and soft candies, pastry decoration, snack, sugar-coated almonds, sugar-coated chocolate candies. *^b^* Number of samples analyzed.

**Table 3:** Instrumental parameters for total titanium analysis.

Parameters	Operating Conditions
Power RF	1550 W
Nebulizer	Esi PFA-LC
Spray chamber	Scott PFA inert kit
Flow nebulizer	0.82 L min^−1^
Makeup gas	0.31 L min^−1^
Peristaltic pump	0.15 rps
Mode	SQ; MS/MS, either on-mass or in mass shift with NH_3_ (22%)
Sampling period	30 s
Integration time	2 s
Selected masses Q1	^46^Ti, ^47^Ti, ^48^Ti, ^49^Ti, ^50^Ti, ^103^Rh
Selected masses Q2	^46^Ti, ^47^Ti, ^48^Ti, ^49^Ti; ^50^Ti; ^46^Ti ⟶ ^148^; ^47^Ti ⟶ ^149^, ^48^Ti ⟶ ^150^, ^49^Ti ⟶ ^151^, ^50^Ti ⟶ ^152^, ^103^Rh

**Table 4:** Instrumental parameters for spICP-MS analysis.

Parameters	Operating Conditions
Power RF	1600 W
Nebulizer	Quartz concentric
Spray chamber	Cyclonic spray chamber
Flow nebulizer	0.99 L min^−1^
Peristaltic pump	−20 rps
Mode	Standard
QID	On
Selected mass	^48^Ti
Dwell time	100 µs
Sampling time	60 s
Transport efficiency	7.51 ± 0.18%
Density	3.8 (g cm^−3^)
Mass fraction TiO_2_/Ti	1.67

**Table 5:** Titanium concentration (µg g^−1^) in the certified reference material and in real food supplement samples as determined following MW-assisted digestion with different acid mixtures. Y/N indicates if E 171 was/was not reported on the item label or if a specific acid was/was not present in the digestion mixture.

					^49^Ti [SQ]	^50^Ti [SQ]	49 ⟶ 49 [MS/MS No Gas]	50 ⟶ 50 [MS/MS No Gas]	47 ⟶ 149 [MS/MS, NH_3_]	48 ⟶ 150 [MS/MS, NH_3_]
Sample	E 171 Labelling	HF	HCl	*n ^a^*	Ti (µg g^−1^) Mean ± SD					
NIST SRM 3280	Y	N	N	*3*	249 ± 72	272 ± 47	264 ± 60	288 ± 56	256 ± 35	237 ± 50
		N	Y	*3*	287 ± 42	400 ± 49	306 ± 41	336 ± 89	378 ± 56	320 ± 58
		Y	N	*3*	4256 ± 34	4270 ± 38	4374 ± 80	4368 ± 74	4119 ± 87	4063 ± 78
		Y	Y	*5*	4054 ± 233	4103 ± 244	4144 ± 236	4158 ± 231	4104 ± 210	4067 ± 193
**Food supplements**										
Melatonin supplement	Y	N	N	*3*	349 ± 114	400 ± 59	459 ± 103	441 ± 45	438 ± 104	447 ± 118
		N	Y	*3*	180 ± 45	172 ± 21	185 ± 72	183 ± 48	188 ± 47	199 ± 55
		Y	Y	*3*	2266 ± 45	2264 ± 37	2256 ± 40	2238 ± 28	2292 ± 38	2260 ± 42
Valerian supplement	Y	N	N	*3*	113 ± 50	85 ± 13	123 ± 11	108 ± 12	209 ± 16	134 ± 23
		N	Y	*3*	286 ± 73	207 ± 60	289 ± 85	291 ± 79	270 ± 95	291 ± 101
		Y	Y	*3*	3567 ± 154	3579 ± 145	3561 ± 180	3537 ± 152	3612 ± 181	3500 ± 174
Soy-isoflavones supplement	Y	Y	Y	*3*	1398 ± 172	1402 ± 158	1388 ± 161	1388 ± 158	1421 ± 170	1413 ± 164
Multi-B supplement	Y	Y	Y	*3*	1979 ± 144	1990 ± 136	1977 ± 132	1979 ± 148	2021 ± 139	1997 ± 135

*^a^* Number of samples analyzed.

**Table 6:** Titanium concentration (µg g^−1^) in food samples as determined following MW-assisted digestion with different acid mixtures. Y/N indicates if E 171 was/was not reported on the item label or if HF was/was not present in the digestion mixture.

				^49^Ti [SQ]	^50^Ti [SQ]	49 ⟶ 49 [MS/MS No Gas]	50 ⟶ 50 [MS/MS No Gas]	47 ⟶ 149 [MS/MS, NH_3_]	48 ⟶ 150 [MS/MS, NH_3_]
Sample	E 171 Labelling	HF	*n ^b^*	Ti (µg g^−1^) Mean ± SD					
Croissant	Y	N	*3*	57 ± 23	51 ± 12	76 ± 12	62 ± 24	79 ± 39	67 ± 17
		Y	*5*	906 ± 303	902 ± 296	904 ± 303	913 ± 308	939 ± 310	911 ± 294
High protein cappuccino	Y	N	*3*	159 ± 126	133 ± 93	177 ± 70	133 ± 74	200 ± 166	180 ± 108
		Y	*3*	3020 ± 43	2999 ± 30	3004 ± 38	3005 ± 16	3072 ± 15	2949 ± 20
Cappuccino powder	N	N	*3*	<0.26 *^b^*	0.27 ± 0.02	<0.25 *^b^*	0.24 ± 0.03	0.09 ± 0.02	0.10 ± 0.01
		Y	*3*	<0.26 *^b^*	0.19 ± 0.10	<0.25 *^b^*	0.17 ± 0.10	0.15 ± 0.09	0.14 ± 0.08
Fair trade chocolate bar	N	N	*3*	2.99 ± 0.52	3.46 ± 0.65	2.78 ± 0.45	3.54 ± 0.61	2.81 ± 0.49	2.78 ± 0.51
		Y	*3*	3.12 ± 0.46	3.48 ± 0.47	2.93 ± 0.44	3.38 ± 0.44	2.74 ± 0.41	2.69 ± 0.38
Dark chocolate chips 1	N	N	*3*	4.16 ± 0.15	5.29 ± 0.29	3.79 ± 0.10	5.15 ± 0.23	3.56 ± 0.11	3.48 ± 0.13
		Y	*3*	5.84 ± 0.86	6.72 ± 0.88	5.47 ± 0.84	6.54 ± 0.89	5.13 ± 0.81	5.09 ± 0.77
White chocolate chips 1	N	N	*3*	<0.26 *^b^*	0.24 ± 0.00	<0.25 *^b^*	0.25 ± 0.00	0.20 ± 0.01	0.19 ± 0.01
		Y	*3*	<0.26 ^a^	0.22 ± 0.17	<0.25 ^a^	0.21 ± 0.16	0.19 ± 0.15	0.18 ± 0.14
Dark chocolate bar 2	N	Y	*3*	2.63 ± 0.42	3.06 ± 0.38	2.39 ± 0.37	2.90 ± 0.39	2.30 ± 0.34	2.29 ± 0.34
Dark chocolate bar 1	N	Y	*3*	2.91 ± 0.17	3.36 ± 0.19	2.60 ± 0.16	3.10 ± 0.17	2.54 ± 0.13	2.52 ± 0.11
White chocolate chips 2	N	Y	*3*	<0.26 *^b^*	<0.07 *^b^*	<0.25 *^b^*	<0.06 *^b^*	<0.04 *^b^*	<0.04 *^b^*
Dark chocolate chips 2	N	Y	*3*	4.21 ± 0.39	4.33 ± 0.40	3.95 ± 0.37	4.22 ± 0.36	3.70 ± 0.32	3.69 ± 0.33
White chocolate bar 1	N	Y	*3*	<0.26 *^b^*	<0.14 *^c^*	<0.25 *^a^*	<0.13 *^b^*	<0.09 *^c^*	0.09 ± 0.01
White chocolate bar 2	N	Y	*3*	<0.26 *^b^*	<0.07 *^b^*	<0.25 *^b^*	<0.06 *^b^*	<0.04 *^b^*	<0.04 *^b^*
Milk and chocolate snack	N	Y	*5*	1.25 ± 0.26	1.39 ± 0.28	1.10 ± 0.24	1.22 ± 0.24	1.17 ± 0.25	1.13 ± 0.24
Vanilla and cocoa dessert	N	Y	*3*	<0.53 *^c^*	0.41 ± 0.06	<0.49 *^c^*	0.40 ± 0.07	0.39 ± 0.06	0.38 ± 0.06
Cream ice-cream	Y	Y	*3*	<0.26 *^b^*	<0.07 *^b^*	<0.25 *^b^*	<0.06 *^b^*	<0.04 *^b^*	<0.04 *^b^*

*^a^* Number of samples analyzed. *^b^* LoD. *^c^* LoQ.

**Table 7:** Recovery (%) of E 171 spiked to one food and one food supplement sample. Y/N indicates if E 171 was/was not reported on the item label or if HF was/was not present in the digestion mixture.

	E 171 Labelling			^49^Ti [SQ]	^50^Ti [SQ]	49 ⟶ 49 [MS/MS No Gas]	50 ⟶ 50 [MS/MS No Gas]	47 ⟶ 149 [MS/MS, NH_3_]	48 ⟶ 150 [MS/MS, NH_3_]
Sample		HF	*n ^a^*	%	%	%	%	%	%
Cappuccino powder	N	N	3	32	33	37	36	37	37
Cappuccino powder	N	Y	6	107	107	106	107	109	110
Probiotics supplement	N	Y	4	108	105	107	106	113	117

*^a^* Number of samples analyzed.

**Table 8:** Main descriptors of the number-based PSDs of TiO_2_ extracted from food and food supplements. Size is expressed as ESD. Y/N indicates if E 171 was reported on the item label.

Sample	E 171 Labelling	Mean Particle Diameter (nm)	Most Frequent Size (nm)	Min Particle Diameter (nm)	Max Particle Diameter (nm)	Particles <100 nm (%)
**Food supplements**						
Multi-B supplement	Y	175	102	42	403	28
Melatonin supplement	Y	158	103	39	364	28
Valerian supplement	Y	169	122	54	363	20
Soy-isoflavones supplement	Y	160	115	55	361	23
**Food**						
Croissant	Y	178	198	35	360	15
Dark chocolate chips 1	N	118	83	50	329	49
Dark chocolate bar 1	N	117	78	49	338	54

## Data Availability

Data are contained within this article and the Supplementary Material.

## Supplementary Materials

The following supporting information can be downloaded at: https://www.mdpi.com/article/10.3390/nano13222957/s1, Table S1. Description of the items, i.e., food supplements and foods, analyzed in this study. Table S2. Selected samples also submitted to mineralization without HF. Table S3. Titanium concentrations (µg g^−1^) obtained in single quad mode (SQ) for the isotopes more affected by spectral interferences. All the samples were submitted to MW-assisted acid digestion with the use of HF. (Y/N indicates if E 171 was reported on the item label). Table S4. Precision, expressed as coefficient of variation (%) for repeatability and reproducibility. Repeatibility was calculated by analyzing 5 times the same sample, i.e., SRM 3280, intra-day. Reproducibility was evaluated in the same manner on three different days. Table S5. LODs (LOQs) achieved, based on the 3σ (6σ) criterion where σ is the standard deviation of the measurement of 20 method blanks. NG means no reaction gas used in MS/MS mode. Table S6. Found total titanium concentration (µg g^−1^) in the reference material NIST 3280 using different amounts of HF in the MW-assisted acid digestion. The recovery was calculated on the reference mass fraction of 5400 ± 300 (µg g^−1^). NG means no reaction gas used in MS/MS mode [48].

## References

[1] Braun J. (1997). Titanium Dioxide: A Review. J. Coat. Technol..

[2] Weir A., Westerhoff P., Fabricius L., Hristovski K., Von Goetz N. (2012). Titanium Dioxide Nanoparticles in Food and Personal Care Products. Environ. Sci. Technol..

[3] Faust J.J., Doudrick K., Yang Y., Westerhoff P., Capco D.G. (2014). Food Grade Titanium Dioxide Disrupts Intestinal Brush Border Microvilli in Vitro Independent of Sedimentation. Cell Biol. Toxicol..

[4] Peters R.J.B., Van Bemmel G., Herrera-Rivera Z., Helsper H.P.F.G., Marvin H.J.P., Weigel S., Tromp P.C., Oomen A.G., Rietveld A.G., Bouwmeester H. (2014). Characterization of Titanium Dioxide Nanoparticles in Food Products: Analytical Methods to Define Nanoparticles. J. Agric. Food Chem..

[5] Yang Y., Doudrick K., Bi X., Hristovski K., Herckes P., Westerhoff P., Kaegi R. (2014). Characterization of Food-Grade Titanium Dioxide: The Presence of Nanosized Particles. Environ. Sci. Technol..

[6] Dudefoi W., Terrisse H., Richard-Plouet M., Gautron E., Popa F., Humbert B., Ropers M.H. (2017). Criteria to Define a More Relevant Reference Sample of Titanium Dioxide in the Context of Food: A Multiscale Approach. Food Addit. Contam. Part. A Chem. Anal. Control Expo. Risk Assess..

[7] Fiordaliso F., Foray C., Salio M., Salmona M., Diomede L. (2018). Realistic Evaluation of Titanium Dioxide Nanoparticle Exposure in Chewing Gum. J. Agric. Food Chem..

[8] Yusoff R., Nguyen L.T.H., Chiew P., Wang Z.M., Ng K.W. (2018). Comparative Differences in the Behavior of TiO_2_ and SiO_2_ Food Additives in Food Ingredient Solutions. J. Nanoparticle Res..

[9] Geiss O., Bianchi I., Senaldi C., Bucher G., Verleysen E., Waegeneers N., Brassinne F., Mast J., Loeschner K., Vidmar J. (2021). Particle Size Analysis of Pristine Food-Grade Titanium Dioxide and E 171 in Confectionery Products: Interlaboratory Testing of a Single-Particle Inductively Coupled Plasma Mass Spectrometry Screening Method and Confirmation with Transmission Electron Microscopy. Food Control.

[10] Verleysen E., Waegeneers N., Brassinne F., De Vos S., Jimenez I.O., Mathioudaki S., Mast J. (2020). Physicochemical Characterization of the Pristine E171 Food Additive by Standardized and Validated Methods. Nanomaterials.

[11] Chen X.X., Cheng B., Yang Y.X., Cao A., Liu J.H., Du L.J., Liu Y., Zhao Y., Wang H. (2013). Characterization and Preliminary Toxicity Assay of Nano-Titanium Dioxide Additive in Sugar-Coated Chewing Gum. Small.

[12] Lim J.H., Bae D., Fong A. (2018). Titanium Dioxide in Food Products: Quantitative Analysis Using ICP-MS and Raman Spectroscopy. J. Agric. Food Chem..

[13] Sungur Ş., Kaya P., Koroglu M. (2020). Determination of Titanium Dioxide Nanoparticles Used in Various Foods. Food Addit. Contam. Part. B Surveill..

[14] Noireaux J., López-Sanz S., Vidmar J., Correia M., Devoille L., Fisicaro P., Loeschner K. (2021). Titanium Dioxide Nanoparticles in Food: Comparison of Detection by Triple-Quadrupole and High-Resolution ICP-MS in Single-Particle Mode. J. Nanoparticle Res..

[15] Verleysen E., Brassinne F., Van Steen F., Waegeneers N., Cheyns K., Machiels R., Mathioudaki S., Jimenez I.O., Ledecq M., Mast J. (2022). Towards a Generic Protocol for Measuring the Constituent Particle Size Distribution of E171 in Food by Electron Microscopy. Food Control.

[16] Al Mutairi M.A., BinSaeedan N.M., Alnabati K.K., Alotaibi A., Al-Mayouf A.M., Ali R., Alowaifeer A.M. (2023). Characterisation of Engineered Titanium Dioxide Nanoparticles in Selected Food. Food Addit. Contam. Part. B Surveill..

[17] More S., Bampidis V., Benford D., Bragard C., Halldorsson T., Hernández-Jerez A., Hougaard Bennekou S., Koutsoumanis K., Lambré C., Machera K. (2021). Guidance on Risk Assessment of Nanomaterials to Be Applied in the Food and Feed Chain: Human and Animal Health. EFSA J..

[18] Younes M., Aquilina G., Castle L., Engel K.H., Fowler P., Frutos Fernandez M.J., Fürst P., Gundert-Remy U., Gürtler R., Husøy T. (2021). Safety Assessment of Titanium Dioxide (E171) as a Food Additive. EFSA J..

[19] European Commission (2022). Commission Regulation (EU) 2022/63 of 14 January 2022; Amending Annexes II and III to Regulation (EC) No 1333/2008 of the European Parliament and of the Council as Regards the Food Additive Titanium Dioxide (E 171). OJ L.

[20] De la Calle I., Menta M., Klein M., Maxit B., Séby F. (2018). Towards Routine Analysis of TiO_2_ (Nano-)Particle Size in Consumer Products: Evaluation of Potential Techniques. Spectrochim. Acta Part. B At. Spectrosc..

[21] De la Calle I., Menta M., Klein M., Séby F. (2017). Screening of TiO_2_ and Au Nanoparticles in Cosmetics and Determination of Elemental Impurities by Multiple Techniques (DLS, SP-ICP-MS, ICP-MS and ICP-OES). Talanta.

[22] Bucher G., Auger F. (2019). Combination of 47Ti and 48Ti for the Determination of Highly Polydisperse TiO_2_ Particle Size Distributions by SpICP-MS. J. Anal. At. Spectrom..

[23] Candás-Zapico S., Kutscher D.J., Montes-Bayón M., Bettmer J. (2018). Single Particle Analysis of TiO_2_ in Candy Products Using Triple Quadrupole ICP-MS. Talanta.

[24] Geiss O., Ponti J., Senaldi C., Bianchi I., Mehn D., Barrero J., Gilliland D., Matissek R., Anklam E. (2020). Characterisation of Food Grade Titania with Respect to Nanoparticle Content in Pristine Additives and in Their Related Food Products. Food Addit. Contam. Part. A Chem. Anal. Control Expo. Risk Assess..

[25] Givelet L., Truffier-Boutry D., Noël L., Damlencourt J.F., Jitaru P., Guérin T. (2021). Optimisation and Application of an Analytical Approach for the Characterisation of TiO_2_ Nanoparticles in Food Additives and Pharmaceuticals by Single Particle Inductively Coupled Plasma-Mass Spectrometry. Talanta.

[26] Bucher G., El Hadri H., Asensio O., Auger F., Barrero J., Rosec J.P. (2024). Large-Scale Screening of E171 Food Additive (TiO_2_) on the French Market from 2018 to 2022: Occurrence and Particle Size Distribution in Various Food Categories. Food Control.

[27] Epstein E. (2000). The Discovery of the Essential Elements. Discoveries in Plant Biology.

[28] WHO Task Group on Environmental Health Criteria for Titanium (1982). Environmental Health Criteria 24: Titanium.

[29] Cornu S., Lucas Y., Lebon E., Ambrosi J.P., Luizao F., Rouiller J., Bonnay M., Neal C. (1999). Evidence of titanium mobility in soil profiles, Manaus, central Amazonia. Geoderma.

[30] Kelemen G., Keresztes A., Bacsy E., Feher M., Fodor P., Pais I. (1993). Distribution and Intracellular Localization of Titanium in Plants After Titanium Treatment. Food Struct..

[31] Lyu S., Wei X., Chen J., Wang C., Wang X., Pan D. (2017). Titanium as a Beneficial Element for Crop Production. Front. Plant Sci..

[32] Silva S., Dias M.C., Silva A.M.S. (2022). Titanium and Zinc Based Nanomaterials in Agriculture: A Promising Approach to Deal with (A)Biotic Stresses?. Toxics.

[33] Rodríguez-González V., Terashima C., Fujishima A. (2019). Applications of Photocatalytic Titanium Dioxide-Based Nanomaterials in Sustainable Agriculture. J. Photochem. Photobiol. C Photochem. Rev..

[34] Madanayake N.H., Adassooriya N.M. (2021). Phytotoxicity of Nanomaterials in Agriculture. Open Biotechnol. J..

[35] Simonin M., Richaume A., Guyonnet J.P., Dubost A., Martins J.M.F., Pommier T. (2016). Titanium Dioxide Nanoparticles Strongly Impact Soil Microbial Function by Affecting Archaeal Nitrifiers. Sci. Rep..

[36] Gogos A., Moll J., Klingenfuss F., Heijden M., Irin F., Green M.J., Zenobi R., Bucheli T.D. (2016). Vertical Transport and Plant Uptake of Nanoparticles in a Soil Mesocosm Experiment. J. Nanobiotechnology.

[37] European Committee for Food Contact Materials and Articles (Partial Agreement) (CD-P-MCA) (2022). Technical Guide on Metals and Alloys Used in Food Contact Materials and Articles, a Practical Guide for Manufacturers and Regulators.

[38] Koller D., Bramhall P., Devoy J., Goenaga-Infante H., Harrington C.F., Leese E., Morton J., Nuñez S., Rogers J., Sampson B. (2018). Analysis of Soluble or Titanium Dioxide Derived Titanium Levels in Human Whole Blood: Consensus from an Inter-Laboratory Comparison. Analyst.

[39] Rompelberg C., Heringa M.B., van Donkersgoed G., Drijvers J., Roos A., Westenbrink S., Peters R., van Bemmel G., Brand W., Oomen A.G. (2016). Oral Intake of Added Titanium Dioxide and Its Nanofraction from Food Products, Food Supplements and Toothpaste by the Dutch Population. Nanotoxicology.

[40] Lomer M.C.E., Thompson R.P.H., Commisso J., Keen C.L., Powell J.J. (2000). Determination of Titanium Dioxide in Foods Using Inductively Coupled Plasma Optical Emission Spectrometry. Analyst.

[41] Sprong C., Bakker M., Niekerk M., Vennemann F. (2015). Exposure Assessment of the Food Additive Titanium Dioxide (E 171) Based on Use Levels Provided by the Industry.

[42] EFSA Panel on Food Additives and Nutrient Sources Added to Food (ANS) (2016). Re-Evaluation of Titanium Dioxide (E 171) as a Food Additive. EFSA J..

[43] Hetzer B., Gräf V., Walz E., Greiner R. (2021). Characterisation of TiO_2_-Containing Pearlescent Pigments with Regard to the European Union Labelling Obligation of Engineered Nanomaterials in Food. Food Addit. Contam. Part. A Chem. Anal. Control Expo. Risk Assess..

[44] Younes M., Aquilina G., Castle L., Engel K.H., Fowler P., Frutos Fernandez M.J., Fürst P., Gürtler R., Gundert-Remy U., Husøy T. (2020). Re-Evaluation of Sodium Aluminium Silicate (E 554) and Potassium Aluminium Silicate (E 555) as Food Additives. EFSA J..

[45] Ferraris F., Raggi A., Ponti J., Mehn D., Gilliland D., Savini S., Iacoponi F., Aureli F., Calzolai L., Cubadda F. (2023). Agglomeration Behaviour and Fate of Food-Grade Titanium Diox-2 Ide in Human Gastrointestinal Digestion and in the Lysosomal 3 Environment. Nanomaterials.

[46] Pace H.E., Rogers N.J., Jarolimek C., Coleman V.A., Higgins C.P., Ranville J.F. (2011). Determining Transport Efficiency for the Purpose of Counting and Sizing Nanoparticles via Single Particle Inductively Coupled Plasma Mass Spectrometry. Anal. Chem..

[47] Balcaen L., Bolea-Fernandez E., Resano M., Vanhaecke F. (2014). Accurate Determination of Ultra-Trace Levels of Ti in Blood Serum Using ICP-MS/MS. Anal. Chim. Acta.

[48] Mech A., Rauscher H., Rasmussen K., Babick F., Hodoroaba V.-D., Ghanem A., Wohlleben W., Marvin H., Brüngel R., Friedrich C.M. (2020). The NanoDefine Methods Manual. Part 3: Standard Operating Procedures (SOPs): JRC117501.

