# Supplementary material for: An ICP-MS-Based Analytical Strategy for Assessing Compliance with the Ban of E 171 as a Food Additive on the EU Market
Source: Nanomaterials (Basel). 2023 Nov 15;13(22):2957. doi: 10.3390/nano13222957 (PMC10674805; doi:10.3390/nano13222957)
Supplement: Supplementary file 1 [file nanomaterials-13-02957-s001.zip › nanomaterials-2692945-supplementary.pdf]

Supplementary information

An ICP-MS-Based Analytical Strategy for Assessing Compliance with the Ban of E 171 as a Food Additive on the EU Market

Francesca Ferraris [1], Carlos Adelantado [2], Andrea Raggi [1], Sara Savini [1,†], Mohammed Zougagh [3,4], Ángel Ríos [3,4] and Francesco Cubadda [1,*]

[1] National Reference Laboratory for Nanomaterials in Food, Department of Food Safety, Nutrition and Veterinary Public Health, Istituto Superiore di Sanità—National Institute of Health, 00161 Rome, Italy; francesca.ferraris@iss.it (F.F.); andrea.raggi@iss.it (A.R.); sara.savini7@gmail.com (S.S.)

[2] Flemish Institute for Technological Research (VITO), 2400 Mol, Belgium; carlos.adelantadosanchez@vito.be

[3] Department of Analytical Chemistry and Food Technology, University of Castilla-La Mancha, 13071 Ciudad Real, Spain; mohammed.zougagh@uclm.es (M.Z.); angel.rios@uclm.es (Á.R.)

[4] Regional Institute for Applied Scientific Research, IRICA, 13005 Ciudad Real, Spain

* Correspondence: francesco.cubadda@iss.it

† Affiliation at time of study.

Materials and Methods

Table S1. Description of the items, i.e. food supplements and foods, analyzed in this study.

Sample	Label
Food Supplements	
Valerian supplement (tablets)	Bulking agent: Microcrystalline cellulose; Valerian (Valeriana officinalis L.) root d.e. tit. (0.4%) Valerenic acid, bulking agent: Calcium phosphate; coating agent: Hydroxypropyl methylcellulose; anti-caking agents: Cross-linked sodium carboxymethylcellulose Silicon dioxide, Vegetable magnesium stearate; dye: Titanium dioxide; anti-caking agent: Vegetable stearic acid; colour: Chlorophyllin-copper complexes
Melatonin supplement (tablets)	Bulking agents: Microcrystalline cellulose; Calcium phosphate; Passion flower (Passiflora incarnata L.) aerial part d.e. tit. (4%) Flavonoids, Valerian (Valeriana officinalis L.) root d.e. tit. (0.4%) Valerenic acid, lemon balm (Melissa officinalis L.) aerial part d.e. tit. (5%) Rosmarinic acid, Freshwater pollen (Eschscholtzia californica Cham.) aerial part d.e. (0.35%) Alkaloids, anti-caking agent: Crosslinked sodium carboxymethylcellulose; coating agent: Hydroxypropyl methylcellulose, anti-caking agents: Silicon dioxide, Vegetable magnesium stearate; colouring: Titanium dioxide anti-caking agent: Vegetable stearic acid; Melatonin, colour: Chlorophyllin-copper complexes
Multi-B supplement (tablets)	Bulking agents: Microcrystalline cellulose, Calcium phosphate; Nicotinamide (vitamin PP), Calcium D-pantothenate (Pantothenic acid), Pyridoxine hydrochloride (Vitamin B6), glazing agent: Hydroxypropyl methylcellulose; Riboflavin (Vitamin B2), Thiamine monohydrate (Vitamin B1), anti-caking agent: Vegetable magnesium stearate; Cyanocobalamin (Vitamin B12), anti-caking agent: Silicon dioxide; dye: Titanium dioxide; anti-caking agent: Vegetable stearic acid; Pteroyl-monoglutamic acid (Folic acid), colours: Yellow iron oxide, Red iron oxide

Soy-isoflavones supplement (tablets)	Calcium carbonate, bulking agent: Microcrystalline cellulose; SOY (Glycine max L.) seeds d.e. tit. (40%) Isoflavones, DL-alpha-tocopheryl acetate (Vitamin E), stabilizer: Polyvinylpyrrolidone, glazing agent: Hydroxypropyl methylcellulose; anti-caking agents: Crosslinked sodium carboxymethyl cellulose, Vegetable magnesium stearate, Silicon dioxide; Pyridoxine hydrochloride (Vitamin B6), Cholecalciferol (Vitamin D3), Cyanocobalamin (Vitamin B12), colour: Titanium dioxide, anti-caking agent: Vegetable stearic acid, Pteroyl-monoglutamic acid (Folate), colour: Iron oxide
Probiotics supplement (powder)	Sweetener: Sorbitol, (from corn or wheat), Fructo-oligosaccharides, Lactobacillus acidophilus R52 (contains milk derivatives), Bifidobacterium lactis BCL-01, anti-caking agent: Silicon dioxide, sweetener: Sucralose

Food	
High protein cappuccino	Milk proteins (emulsifier: Sunflower lecithin), Skimmed milk powder (10.7%), Soluble coffee (6%), Low-fat cocoa, powder (2.6%), Flavouring, Wheat fibres, Salt, Vegetable oil (Palm oil), anti-caking agents: Potassium and Magnesium carbonate, thickeners: Guar and Xanthan gum and Locust bean gum, colour: Titanium dioxide, Vitamins (C, B3, E, B5, B6, B2, B1, A , B9, B8, B12), Maltodextrin, Lactose, sweeteners: Sucralose. optional statement (processing aid): Silicon dioxide [nano] Contains: Milk, Gluten (Wheat)
Croissant	Soft wheat flour, Fresh milk cream (25%) [Glucose syrup, Sugar, Water, Pasteurized fresh milk (10% in the cream, corresponding to 2.5% of the total), Vegetable palm fat, Skimmed milk powder, Flavourings, thickeners: Pectin, colourings: E171, preservatives: Potassium sorbate], Vegetable margarine (Palm fat, Water, Sunflower oil), Sugar, Fresh eggs (4.8%), Natural yeast (Gluten), Cereal flour (2.5%) (Oats, rice, Barley, Spelled, Buckwheat), emulsifiers: Mono-and diglycerides of fatty acids, Skimmed milk powder, Salt, Flavourings, Malted barley flour. May contain traces of nuts and soy
Dark chocolate chips 1	Cocoa paste, Sugar, Cocoa butter, emulsifier: Lecithins (soy). Cocoa (48%) minimum. May contain traces of milk.
Dark chocolate chips 2	Cane sugar, Cocoa paste, Cocoa butter, emulsifier: Lecithins (soy), Natural Vanilla flavouring
Fair trade chocolate bar	Dark chocolate coating: Cane sugar, Cocoa paste, Cocoa butter, emulsifier: Soy Lecithin, Natural Vanilla flavour. Cocoa (48%) min. Orange flavored cream filling: Cane sugar, Vegetable oil and fats (Cocoa butter and Sunflower oil), Whey, Lactose, Skimmed milk powder, Emulsifier: Soy Lecithin, Flavouring
Dark chocolate bar 1	Cocoa paste, Sugar, Cocoa butter, Low fat cocoa, Natural flavor. Cocoa (72%) minimum
Dark chocolate bar 2	Cocoa paste, Cane sugar, Cocoa butter, Emulsifiers: Soy Lecithin, Ground Vanilla, *Organic, may contain traces of Nuts, Lactose and Milk proteins, Cocoa, cane sugar, vanilla (99.6%)
Milk and chocolate snack	Sugar, Vegetable oils and fats (cocoa butter, sunflower oil, coconut oil), Type 0 soft wheat flour, Glucose-fructose syrup, Eggs, Milk powder 6.1%, Skimmed milk 5.5%, Skimmed cocoa powder 4.1%, Milk 3.6%, Skimmed milk powder 2.5%, Butter, Milk proteins, Flavourings, Emulsifiers: Mono- and Diglycerides of fatty acids, Soy Lecithin, raising agents: Acid carbonate sodium, Ammonium hydrogen carbonate, Diphosphates, Salt, thickener: Pectin, may contain traces of Hazelnuts and other Nuts

Cream ice-cream	Rehydrated skimmed milk, Glucose-fructose syrup, Sugar, Coconut oil, Concentrated butter, Glucose syrup, Cream (3.5%), Lactose and Milk proteins, Skimmed cocoa powder, Cocoa mass, Emulsifiers (mono- and diglycerides of fatty acids, ammonium phosphatides, SOY lecithin), Thickeners (Locust bean gum, Guar gum, Carrageenan), Flavourings, Cocoa butter, Colors (Iron oxide and hydroxide, Titanium dioxide), Glazing agents (Shellac)
White chocolate chips 1	Cane sugar, Whole milk powder (24%), Cocoa butter (21%), Skimmed milk powder, Emulsifier Lecithins (Soy), Natural vanilla flavor
White chocolate chips 2	Sugar, Whole milk powder, Cocoa butter, Skimmed milk powder, Emulsifier Lecithins (Soy), Natural vanilla flavor
White chocolate bar 1	Sugar; Cocoa butter; Powdered milk; Emulsifier: Lecithins (Soy)
White chocolate bar 2	Cane sugar, Cocoa butter, Milk powder (28%), Emulsifiers: Lecithins (Soy), Ground vanilla, Organic, may contain traces of Nuts, Cocoa, cane sugar, Vanilla (71.5%)
Vanilla and cocoa dessert	Glucose syrup, Skimmed milk powder, Lactose and Milk proteins, Emulsifiers: Mono- and Diglycerides of fatty acids, Thickeners: sodium alginate, Gelatin, Natural vanilla flavor (0.07%), Flavourings, may contain traces of Egg and Nuts, Rehydrated skimmed milk, Skimmed cocoa coating (11%) (Vegetable oils (Coconut oil, Sunflower oil), Sugar, Skimmed cocoa powder (15.9%), emulsifier: Lecithins (Soy)) , Sugar, Coconut oil
Cappuccino powder	Skimmed milk powder (31%), Sugar (26%), Glucose syrup, Instant coffee (14%), Coconut fat, Stabiliser: sodium phosphate

Table S2. Selected samples also submitted to mineralization without HF.

Sample name	E 171 labelling
Valerian supplement	Y
Melatonin supplement	Y
High protein cappuccino	Y
Croissant	Y
Dark chocolate chips 1	N
Fair trade chocolate bar	N
White chocolate chips 1	N
Cappuccino powder	N

Results

Table S3. Titanium concentrations (μg g^{-1}) obtained in single quad mode (SQ) for the isotopes more affected by spectral interferences. All the samples were submitted to MW-assisted acid digestion with the use of HF. (Y/N indicates if E 171 was reported on the item label).

			^{46}Ti [SQ]		^{47}Ti [SQ]		^{48}Ti [SQ]	
**Sample**	**E 171 reported on the label**	*n*	Mean	SD	Mean	SD	Mean	SD
NIST SRM 3280	Y	*3*	4242	69	4425	36	4292	21
**Food supplements**								

Melatonin supplement	Y	*3*	2173	125	2335	34	2440	86
Valerian supplement	Y	*3*	3572	140	3611	134	3773	132
Soy-isoflavones supplement	Y	*3*	1352	152	1380	161	1465	150
Multi-B supplement	Y	*3*	1876	190	2073	148	2013	130
**Food**								
Croissant	Y	*5*	881	303	900	294	918	299
High protein cappuccino	Y	*3*	3075	93	3051	63	3096	50
Cappuccino powder	N	*4*	2.87	0.28	11.55	0.91	17.81	0.86
Fair trade chocolate bar	N	*3*	4.29	0.56	7.23	0.65	6.93	0.62
Dark chocolate chips 1	N	*3*	6.70	0.88	13.05	1.13	9.40	1.15
White chocolate chips 1	N	*3*	4.20	1.46	8.24	0.98	12.35	1.23
Dark chocolate bar 2	N	*3*	3.14	0.34	10.46	0.33	6.10	0.49
Dark chocolate bar 1	N	*3*	3.29	0.19	10.98	0.96	6.05	0.35
White chocolate chips 2	N	*3*	4.25	1.42	8.09	0.96	11.28	0.92
Dark chocolate chips 2	N	*3*	4.44	0.43	10.26	0.61	6.82	0.45
White chocolate bar 1	N	*3*	2.41	0.14	6.02	0.91	10.85	0.92
White chocolate bar 2	N	*3*	2.31	0.05	7.31	0.44	12.12	0.21
Milk and chocolate snack	N	*5*	4.81	0.76	9.45	1.33	8.85	0.78
Vanilla and cocoa dessert	N	*3*	3.21	0.42	4.63	0.33	6.05	0.45
Cream ice-cream	Y	*3*	2.81	1.16	4.86	1.23	7.85	1.37

Table S4. Precision, expressed as coefficient of variation (%) for repeatability and reproducibility. Repeatability was calculated by analyzing 5 times the same sample, i.e. SRM 3280, intra-day. Reproducibility was evaluated in the same manner on three different days.

	**^{49}Ti [SQ]**	**^{50}Ti [SQ]**	**49 -> 49 Ti [MS/MS No Gas]**	**50 -> 50 Ti [MS/MS No Gas]**	**47 -> 149 Ti [MS/MS, NH_3]**	**48 -> 150 Ti [MS/MS, NH_3]**
**Repeatability**						
	1.2	1.1	0.8	0.3	1.1	1.1
**Reproducibility**						
	1.1	2.0	2.1	1.1	2.9	3.0

Table S5. LODs (LOQs) achieved, based on the 3σ (6σ) criterion where σ is the standard deviation of the measurement of 20 method blanks. NG means no reaction gas used in MS/MS mode.

	**^{49}Ti [SQ]**	**^{50}Ti [SQ]**	**49 -> 49 Ti [MS/MS NG]**	**50 -> 50 Ti [MS/MS NG]**	**47 -> 149 Ti [MS/MS, NH_3]**	**48 -> 150 Ti [MS/MS, NH_3]**
			Ti (μg g^{-1})			
LOD	0.26	0.07	0.25	0.06	0.04	0.04
LOQ	0.53	0.14	0.49	0.13	0.09	0.08

Table S6. Found total titanium concentration (µg g^{-1}) in the reference material NIST 3280 using different amounts of HF in the MW-assisted acid digestion. The recovery was calculated on the reference mass fraction of 5400 ± 300 (µg g^{-1}). NG means no reaction gas used in MS/MS mode [48].

	^{49}Ti [SQ]			^{50}Ti [SQ]			49 -> 49 Ti [MS/MS NG]			50 -> 50 Ti [MS/MS NG]			47 -> 149 Ti [MS/MS, NH_3]			48 -> 150 Ti [MS/MS, NH_3]		
	Ti (µg g^{-1})																	
**HF (mL)**	**Mean**	**SD**	**Rec. (%)**	**Mean**	**SD**	**Rec. (%)**	**Mean**	**SD**	**Rec. (%)**	**Mean**	**SD**	**Rec. (%)**	**Mean**	**SD**	**Rec. (%)**	**Mean**	**SD**	**Rec. (%)**
0.75	4222	110	78	4190	95	78	4185	99	77	4235	123	78	4468	83	83	4529	89	84
1.00	4348	317	81	4323	318	80	4360	254	81	4433	262	82	4578	287	85	4617	307	85
1.25	4665	132	86	4637	117	86	4624	107	86	4695	100	87	4885	174	90	4922	186	91
1.50	4547	177	84	4525	159	84	4524	138	84	4579	187	85	4750	203	88	4789	156	89
